# Glutamine Synthetase: Localization Dictates Outcome

**DOI:** 10.3390/genes9020108

**Published:** 2018-02-19

**Authors:** Alessandra Castegna, Alessio Menga

**Affiliations:** 1 Department of Biosciences, Biotechnologies and Biopharmaceutics, University of Bari, 70125 Bari, Italy; 2 Hematology Unit, National Cancer Research Center, Istituto Tumori ‘Giovanni Paolo II’, 70124 Bari, Italy; mengaalessio@gmail.com

**Keywords:** glutamine, glutamine synthetase, M2 macrophages, adipocytes, immunosuppressive, glutaminase, cancer, brain, metabolism, immunometabolism

## Abstract

Glutamine synthetase (GS) is the adenosine triphosphate (ATP)-dependent enzyme that catalyses the synthesis of glutamine by condensing ammonium to glutamate. In the circulatory system, glutamine carries ammonia from muscle and brain to the kidney and liver. In brain reduction of GS activity has been suggested as a mechanism mediating neurotoxicity in neurodegenerative disorders. In cancer, the delicate balance between glutamine synthesis and catabolism is a critical event. In vitro evidence, confirmed in vivo in some cases, suggests that reduced GS activity in cancer cells associates with a more invasive and aggressive phenotype. However, GS is known to be highly expressed in cells of the tumor microenvironment, such as fibroblasts, adipocytes and immune cells, and their ability to synthesize glutamine is responsible for the acquisition of protumoral phenotypes. This has opened a new window into the complex scenario of the tumor microenvironment, in which the balance of glutamine consumption versus glutamine synthesis influences cellular function. Since GS expression responds to glutamine starvation, a lower glutamine synthesizing power due to the absence of GS in cancer cells might apply a metabolic pressure on stromal cells. This event might push stroma towards a GS-high/protumoral phenotype. When referred to stromal cells, GS expression might acquire a ‘bad’ significance to the point that GS inhibition might be considered a conceivable strategy against cancer metastasis.

## 1. Introduction

Glutamine is the most abundant amino acid in mammalian blood, making up as much as 20% of the total amino acid content [1]. Glutamine is essential for protein and aminoacid synthesis via transamination and purines, pyrimidines, glucosamine and carbamoyl phosphate synthesis [2]. As the glutamine coming from diet is metabolized in the intestine, most of the body glutamine is synthesized de novo. The only enzymatic activity able to do so is glutamine synthetase (GS) (EC 6.3.1.2), an adenosine triphosphate (ATP)-dependent enzyme that catalyzes the formation of glutamine from glutamic acid and ammonia [3]. GS is a key regulator of nitrogen metabolism since it reduces free ammonia by converting it into glutamine, which enters the blood stream and is transported to the liver [4,5,6]. In this way glutamine controls the uptake of nitrogen where required (e.g., for nucleotide and nitrogen-rich aminoacids synthesis) and its removal where accumulated, reducing its toxicity. For this reason, blood concentration of glutamine is very high compared to other amino acids. Intestinal bacteria produce a high amount of ammonia through the action of urease [7]. Additionally, degradation of proteins and aminoacids contributes to ammonia accumulation, the physiological concentration of which in the blood reaches 35 μmol/L. 

GS structure has been extensively studied and its function elucidated in different body tissues [8]. GS is ubiquitously present in brain, liver, muscle, adipose tissue and lung [9]. In the liver, glutamine is taken up by the periportal cells in which ammonia is released to be incorporated into urea. Periportal cells are unable to synthesize glutamine, whereas perivenous cells express high GS levels. In this way periportal and perivenous cells act in the opposite way, by removing and releasing glutamine, respectively. The liver thus acts as a rheostat for homeostatic control of blood glutamine and ammonia [10]. The importance of liver GS is underlined by the effects of the specific deletion of GS in mouse liver, which are hyperammonemia, oxidative stress in brain tissue, behavior abnormalities, cognitive and motor deficits [11]. Ammonia accumulation is also exhibited by the muscle-specific GS deletant mouse [12]. Lack of GS function due to point mutation on GS gene leads to a fatal condition associated to multiorgan failure [13]. All these studies point to a crucial role of GS as a multitask supporter of the fundamental functions mentioned above.

Recent insights into the role of GS in modulating immune function [14,15] have opened a totally new scenario in cancer, in which GS activity might represent paradoxically a disadvantage. Although muscle and liver GS are functionally crucial for sustaining the above-mentioned functions, in this review we will focus on GS function with respect to brain physiology and cancer development, in which evidence is supporting newly discovered functional roles of glutamine synthesis. We are just now beginning to understand it.

## 2. GS in Brain Physiological and Pathological Conditions

The brain is particularly susceptible to ammonia toxicity, due to two specific reasons: (i) the urea cycle does not occur in the brain; (ii) ammonia can easily cross the blood brain barrier. For these reasons, it relies on the reaction catalyzed by GS, which takes place mainly in astrocytes, as a main instrument for ammonia removal [16]. The role of astrocytes in ammonia detoxification is further emphasized by their anatomical proximity with the blood-brain barrier [16]. The perivascular astrocyte end-feet, which surrounds the abluminal domain of microvessel-associated endothelial cells, represents a metabolic pool buffering the blood content with respect to the rest of the brain. In this way, blood releases ammonia to the astrocytic compartment to be metabolized by GS, reaction that prevents further progression of the harmful molecule into the neuronal compartment [17]. 

Additionally, GS is an enzyme of crucial neurochemical importance, since it converts the neurotransmitter L-glutamate into L-glutamine [18]. L-glutamate is incorporated into vesicles at the neuronal synaptic junction and is released upon stimulation. After its function is completed, glutamate is then taken up by astrocytes where it is converted into L-glutamine and recycled back into the neuron vesicles, where it may be re-converted back to glutamate [18]. In this cycle, glutamate is not metabolized but just recycled in order to avoid excitotoxic events, described in many diseases [19], due to overstimulation of neurons by excessive building up of the aminoacid. Brain GS plays a main role in the glutamine-glutamate-γ-aminobutyric acid (GABA) cycle, balancing excitatory and inhibitory synaptic transmission through synthesis, cellular release, and extracellular uptake of glutamate and GABA [20].

The role of brain GS has been highlighted in neurodegenerative disorders, in which a decline in GS activity has been noted due to oxidation-related loss of function [21]. Indeed, due to its particular sensitivity to inactivation by oxidant agents [22,23,24,25], GS activity has been considered an indicator of the harmful action of reactive oxygen species (ROS), leading to brain damage. In neurodegenerative conditions such as Alzheimer disease (AD) brain GS activity is reduced compared to age-matched healthy controls [25,26,27]. A similar decline in GS activity has been noted during aging [26]. Further studies on GS at a molecular level evidenced increased protein oxidative modifications in AD brain, confirming the susceptibility of GS to oxidative stress and the important role of GS oxidation in contributing to AD neurodegeneration [28,29]. This statement is also substantiated by the documented inverse correlation between the amount of GS in AD brain and the number of amyloid plaques [30] and the increased glutamate/glutamine ratio in AD cerebrospinal fluid [31,32]. Additionally, the glutamate transporter is oxidatively modified [33] and dysfunctional [34] in AD. All these data strongly support the relationship between oxidative modifications and decreased GS activity in AD brain.

The role of GS was investigated in the experimental allergic encephalomyelitis (EAE), experimental murine model of multiple sclerosis. Since pathological changes occur in areas of central nervous system (CNS) tissue remote from inflammatory lesions in EAE mice, we tried to ascertain whether oxidative stress could impair GS function in cortex tissue. Two-dimensional oxyblots and mass spectrometry-based protein fingerprinting identified GS as a specific target of oxidation. Oxidation of GS is associated with a reduction in enzyme activity and increased glutamate/glutamine levels [35]. The possibility that this may cause neurodegeneration through glutamate excitotoxicity is supported by evidence of increased Ca^2+^ levels in cortex extracts from EAE animals with greater disease severity [35]. All these data clearly point to a causative link between GS oxidation and the decline of GS activity in EAE, suggesting that oxidative stress occurs in brain areas that are not actively undergoing inflammation or before inflammation develops in EAE. This event might promote a neurodegenerative process due to the susceptibility of GS to oxidative inactivation.

**Figure 1:**
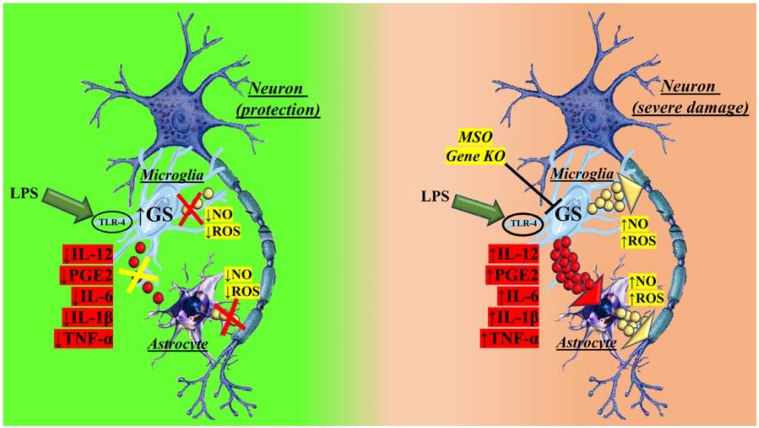
Role of glutamine synthetase (GS) in brain physiology. Brain relies on the reaction catalyzed by GS, which is known to take place mainly in astrocytes, as a fundamental mechanism for ammonia and glutamate removal. Microglia also express GS and participate with astrocytes in this task. Microglia possess an endogenous mechanism modulating their response to a proinflammatory agent, such as lipopolysaccharides (LPSs). By expressing GS microglial response to LPS is controlled, limiting thus the consequent harmful effects on surrounding cells (left). When this mechanism is lost (GS blockade, oxidation-related GS loss of function, GS inhibition with methionine sulfoximine, MSO) microglia engage a strong inflammatory response to LPS, producing inflammatory mediators and effectors, and leading to neuronal damage. ROS: reactive oxygen species; KO: knock-out; IL-12: interleukin-12; IL-6: interleukin-6; IL-1β: interleukin-1β; PGE2: Prostaglandin E2; TNF-α: tumor necrosis factor-alpha; TLR-4: Toll-like receptor 4.

This first study unraveling the role of GS in an inflammatory condition prompted research toward the understanding the role of GS during inflammatory response, which was also strongly suggested by the evidence documenting GS expression in both macrophages and microglia [36]. In particular, brain microglia express the glutamate scavenging system similarly to astrocytes, also known as the cellular glutamate transporter 1 (GLT-1) and GS [36,37,38] in both physiological and pathological conditions [39,40,41]. The presence of this system makes microglia active as astrocytes in protecting neurons by scavenging glutamate. Additionally, glutamate uptake from the extracellular spaces might favour glutathione (GSH) production through the coexpression of GLT-1 and the cystine/glutamate antiporter [42,43,44]. In microglial cells the glutamate conversion to glutamine can modulate the cellular response to an inflammatory stimulus [15]. Basal microglia express low levels of cluster of differentiation 45 (CD45) and major histocompatibility complex class I and II antigens and are maintained in a constant state of relative inactivity. This could be ascribed to the immunomodulatory signals provided by neurons [45] and astroglia [46,47,48,49], together with the protective role of the blood-brain barrier [50]. Microglia can metabolically control their own response to a proinflammatory stimulus by converting glutamate to glutamine [15]. Indeed, when GS is pharmacologically (in vitro, with methionine sulfoximine, MSO) or genetically (in vivo) blocked, microglia increase production and release of inflammatory mediators and effectors following a proinflammatory stimulus, ultimately leading to greater neuronal oxidative stress and injury (Figure 1). 

These results provide the evidence that GS sensitivity to redox balance might represent a strategy by which several mechanisms relevant to the inflammatory response are modulated [15]. Additionally, the beneficial role of GS in brain is not restrained to its ability to remove ammonia and glutamate, but also to maintain microglia in an immunosuppressive state by means of the metabolic reaction catalyzed by the enzyme (Figure 1).

Recently, the relationship between neurodegeneration and brain metabolism has been highlighted in neurodegenerative disorders, particularly in AD. The clinical outcome of AD is likely dependent on deregulation of neuronal metabolism, which is mainly represented by a deficit in glucose utilization seen in AD patients. Recent evidence suggests that neuroinflammation might represent a new form of brain metabolic stress. Inflammatory mechanisms might play a role in synapse and cognitive impairments, by mechanisms leading to effects similar to insulin resistance and glucose intolerance in peripheral tissues [51,52,53,54]. Based on the above considerations, an intriguing connection between AD and diabetes has been postulated. The metabolic abnormalities linked to inflammation, insulin resistance and endoplasmic reticulum (ER) stress are the typical features of glucose intolerance and type 2 diabetes mellitus (T2DM) in peripheral tissues [55]. These very same derangements have been described in AD patients, as AD brains present several markers of insulin resistance, inflammation and ER stress [56,57,58,59,60]. Similarly to adipocytes [61], microglia reduce their insulin-related glucose uptake when GS in inhibited [15]. This result might shed light into one of the unifying mechanisms controlling insulin resistance, inflammation, and metabolism. Microglial interleukin-1 (IL-1), interleukin-6 (IL-6), and tumor necrosis factor-alpha (TNF-α) are known activators of insulin receptor sustrate-1 (IRS-1) serine kinases (IκB kinase, c-Jun N-terminal kinase, and extracellular signal-regulated kinase-2), which decrease IRS-1 activity by phosphorylation [57] as seen in AD [58]. Insulin is known to antagonize the deleterious effects of oxidative stress in the CNS. By stimulating glucose uptake insulin rebalances intracellular ATP levels, reduces ROS formation [62], and, more importantly, reduces glutamate excitotoxicity by decreasing its accumulation in the extracellular milieu [63]. GS activity might then unify different mechanisms through its control of inflammation, insulin resistance, and glutamate excitotoxicity. In light of these new findings, the beneficial effect of GS expression is magnified.

## 3. Glutamine Synthetase in Tumors

Different mammalian organs exhibit distinct modes of glutamine metabolism [64]. For example, kidneys utilize glutamine for pH homeostasis through ammonia and for renal gluconeogenesis [1], whereas lungs, skeletal muscles, and adipose tissues display de novo glutamine synthesis through GS [65]. Similarly, human tumors display a variety of metabolic phenotypes depending on the tissue of origin and the cancer subtype. Within a tumor, different levels of oxygen [66] and perfusion play a role. Additionally, specific transcription factors or oncogenes can drive glutamine metabolism toward divergent directions. Finally, the ability to synthetize glutamine varies not only among different tumor cells but also within cells belonging to the tumor microenvironment (TME). In this very complicated scenario, we will discuss the significance of GS expression.

### 3.1. Glutamine Synthetase in Cancer Cells

Even among tumors that are generated in a specific organ, different cancer subtypes display distinct patterns of glutamine metabolism [64]. Some cancer cells can synthesize glutamine de novo due to GS activity. In other cases, cancer cells rely on extracellular glutamine, which enters mitochondrial metabolism via conversion to glutamate through glutaminase (GLS) [67] followed to 2-oxoglutarate production by glutamate dehydrogenase or aminotransferase [68]. The relationship between GLS and GS is very important in cancer cells and several examples provide clues with this respect [64]. GS expressing cancer cells are more prone to autophagy [69] similarly to B lymphocytes [70]. Luminal breast cancer cells are resistant to a glutamine-less environment due to their ability to synthetize glutamine through GS expression [71], to the point that the produced glutamine is also secreted into the medium [71]. In contrast, basal breast cancer cells, which are sensitive to glutamine deprivation, do not express GS. This specific metabolic feature associates basal breast cancer to a more aggressive and therapy-resistant phenotype compared to luminal breast cancer [71]. This finding has been noted also in melanoma [72] and ovarian cancer cells [73]. Cytotoxic T lymphocyte (CTL) killing sensitive melanoma cells (ME15S) express high levels of GS, whereas the CTL killing resistant cells (ME15R) do not [72]. Similarly, low-invasive ovarian cancer (OVCA) cells (OVCAR3, IGROV1 and OVCA429) highly express GS, compared to high-invasive OVCA cells (SKOV3, SKOV3ip and Hey8), which do not [73]. According to these findings, the absence of GS, associated to high GLS activity, makes cancer cells addicted for glutamine and might associate with a more invasive, aggressive and resistant phenotype. This is confirmed in vivo, as in breast basal and liver tumors [64]. GLS messenger RNA (mRNA) expression, which could be indicator of cancer glutamine addiction, is higher in myeloma [74] and other tumor types, such colon, liver, stomach, and thyroid compared to the surrounding healthy tissue [64] and its blockade is known to hamper in some cases tumor progression [75,76,77]. However, there are examples in which cancer cells express high levels of GS gene [64]. Human glioblastoma multiforme (GBM) tumors display high GS and low GLS activity, and for this reason accumulate large pools of glutamine from glucose [78]. This metabolic feature associates with increased de novo purine biosynthesis and makes GBM cells resistant to glutamine deprivation [67]. GS gene expression has been associated with poor prognosis in breast cancer patients [68,79,80]. Knockdown of GS inhibits SK-BR-3 proliferation (a human epidermal growth factor receptor 2-enriched breast cancer cell line) and abrogates p38 mitogen-activated protein kinase (MAPK) and extracellular signal-regulated kinase 1/2 (ERK1/2) signaling pathways [79]. In contrast with these findings, GS expressing hepatocellular carcinoma (HCC) cells become resistant to sorafenib following GS inhibition and this is related to the tendency of GS expressing HCC cells to undergo autophagy [69]. Furthermore, high GLS activity does not always mirror lower glutamine synthetic capacity, as in the case of non-small cell lung cancer (NSCLC), in which the expression levels of GLS and GS are both high [81]. This might indicate that glutamine synthesis, upregulated in some cancers, represents a metabolic advantage while dealing with glutamine deprivation, noticed both in cell culture and when grown as xenograft tumors in vivo [71,82]. Furthermore, ammonia incorporation into glutamine might represent an advantage for improving cell survival and persistence against ammonia stress, as demonstrated by Kitajima and coworkers [83]. The real picture of glutamine metabolism within the tumor is complicated even further. Metabolic heterogeneity can arise among different regions within a tumor. For example, in highly perfused regions of NSCLC tumors glutamine enters the tricarboxylic acid (TCA) cycle, whereas in less perfused regions glutamine is synthesized from glucose [84]. Furthermore, there are many other factors regulating glutamine metabolism, such as the activity of c-Jun [85], retinoblastoma protein (pRb) [86,87], peroxisome proliferator-activated receptor gamma coactivator 1-alpha (PGC-1α)/estrogen-related receptor-alpha (ERRα) [88], GS acetylation levels [89], and others [90,91]. Different oncogenes can drive development of tumors arising within the same tissue, producing thus metabolically divergent patterns. For example, MET-induced liver tumors loose GLS expression, overexpress GS gene and accumulate glutamine [81]. MYC-driven liver tumors exhibit elevated glutamine catabolism with increased GLS and reduced GS expression, relative to surrounding tissue [77,81]. However, MYC itself can paradoxically induce GS expression by means of an epigenetic mechanism involving GS promoter demethylation [92].

What can be comprehensively inferred from the above findings is that GS expression in cancer cells can vary depending on many different mechanisms, even within the same tumor. In some cases, lack of glutamine synthesis in cancer cells associates with a more invasive, aggressive and resistant phenotype (Table 1). The significance of this event lays probably with the metabolic stress cancer cells transmit to the surrounding cells in virtue of their dependency on external glutamine availability. For this reason, it is fundamental to expand the scenario to other key players within the TME, including stromal cells, in order to obtain a clear picture of the complexity of glutamine metabolism within the tumor.

### 3.2. Glutamine Synthetase in the Tumor Microenvironment

The metabolic phenotype of a given tumor seems to be also crucial for the TME, which consists of different cell types, such as immune cells, fibroblasts, endothelial cells, adipocytes and others. It is evident that the different components of the TME play significant roles in the process of cancer initiation, progression and invasion [93]. Our recent studies highlight and confirm the important role of macrophages as key player within the TME. We show that GS expression is a typical feature of interleukin-10 (IL-10) stimulated- M2-like human macrophages (immunosuppressive and proangiogenic), and pharmacological inhibition of GS skews M2-polarized macrophages toward the M1-like phenotype (pro-inflammatory and antitumoral), characterized by a specific metabolic signature and by hypoxia-inducible factor 1-alpha (HIF1α) stabilization. GS-inhibited macrophages recruit T-cell less efficiently, reduce T-cell suppressive potential, and impair ability to foster endothelial cell branching and cancer cell motility. Genetic ablation of macrophagic GS in tumor-bearing mice inhibits metastasis [14]. These data identify GS activity as crucial mediator of the proangiogenic, immunosuppressive and pro-metastatic function of M2-like macrophages and highlight the possibility to target this enzyme in the treatment of cancer metastasis [14]. 

GS expression is known to be a sensitive indicator of nutrient deprivation as its expression level is under the control of the starvation sensing transcription factor forkhead box O3a (FOXO3A) [70]. At a protein level, GS is post-translationally stabilized in glutamine deprived conditions [89]. In human macrophages nutrient deprivation is sufficient to skew resting cells (M0) toward an M2-like phenotype without any further cytokine treatment [14], in line with previous findings [94]. More interestingly, GS expression is elevated in both M2-polarized and M0 macrophages incubated under conditions of glutamine deprivation, compared to the same cells incubated in glutamine rich media, and this leads to increased extracellular levels of the aminoacid. This indicates that in a glutamine-less environment GS expressing macrophages might secrete glutamine for other cells’ use [14]. The importance of GS in the TME has been also highlighted in an elegant study in ovarian carcinoma mouse models in which the reliance of cancer cells on stromal cancer associated fibroblasts (CAF) metabolism is clearly demonstrated [95]. These fibroblasts, due to the metabolic stress applied by cancer cells, upregulate GS and glutamine synthesis and the secreted glutamine sustains tumor growth [95]. In both these studies, GS expressed in the cells from the TME is exploited metabolically to produce a functional outcome relevant for tumor and metastasis. Similar results are obtained with adipocytes, that are able to secrete glutamine in a GS-dependent fashion when cocultured with pancreatic intraepithelial neoplasia/pancreatic ductal adenocarcinoma (PanIN/PDAC) [96] and with leukemia cells [97].

The importance of GS is confirmed in T-cells, key players of the adaptive immune system within the TME. Cancer cells are known to compete with T cells for nutrients within the TME and their limiting availability suppresses T cell activation and proliferation [98,99,100,101]. Furthermore, glutamine starvation favors the balance between immunocompetent Thelper1 (T_H_1) and immunosuppressive regulatory T cells (T_reg_) functions toward that of a T_reg_ phenotype [102]. GS activity plays a role in that. Human T cell activation in conditions of glutamine deprivation results in CD4^+^T cells with high expression of the T_reg_ transcription factor Forkhead box P3 (FOXP3) and this functional reprogramming is abolished by blocking GS. This suggests that FOXP3^hi^ cells rely on GS to sustain their immunosuppressive activity under limiting availability of extracellular glutamine [103]. All these findings consistently support the role of GS-driven glutamine synthesis in cells of the TME as a fundamental mechanism to provide glutamine in the extracellular milieu and, at the same time, as a key metabolic node controlling immune cell function in a protumoral direction. 

## 4. Conclusions

**Figure 2:**
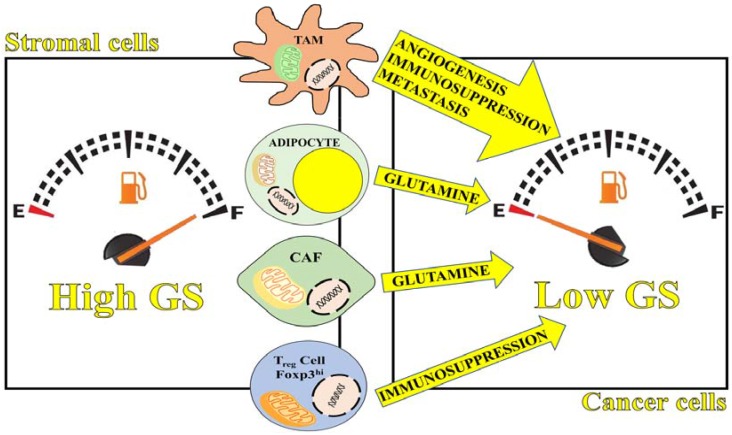
GS expression in cancer versus tumor microenvironment (TME) cells. Reduced ability to synthesize GS, displayed by some cancer cells, might significantly modify the composition of the extracellular milieu in terms of nutrients availability (such as glutamine). Based on the evidence that starvation increases GS levels (by means of forkhead box O3 (FOXO3A) and post-translational protein stabilization), glutamine depletion induced by glutamine dependent-cancer cells might trigger GS expression in cancer associated fibroblasts (CAFs), macrophages, adipocytes and T cells. In these cells glutamine synthesis is known to mediate a metabolic and functional reprogramming. In this way, the inability to synthesize glutamine (reduced GS expression) displayed by some cancer cells might be one of the elements capable of inducing a metabolic pressure on the TME, eventually reprogramming CAFs, adipocytes and immune cell function toward a protumoral phenotype. TAM: tumor-associated macrophages; T_reg_: Regulatory T cells.

All these novel findings point to a protumoral role of GS within the TME. Unexpectedly, the beneficial role of glutamine synthesis in our body can be exploited by cancer cells and manipulated to sustain cancer development. From the analysis of the recent literature it is clear that the significance of GS expression within cancer cells is not straightforward. GS gene expression varies in different tumors and within a tumor. In many cases, the absence of GS is an indicator of glutamine addiction and associates to invasive and aggressive phenotypes. However, the demonstrated role of GS in cells of the TME, such CAFs, macrophages, adipocytes and T cells, together with the intrinsic property of GS gene to respond to conditions of glutamine starvation, might shed light into the significance of GS gene expression within cancer cells. Although this cannot be applied to each tumor type, we suggest that absence or lower GS activity compared to GLS activity in cancer cells, within the whole tumor or locally in specific regions of it, might represent one of the mechanisms by which cancer cells apply a metabolic pressure on the TME cells (Figure 2). 

Since GS gene expression and protein stability respond to glutamine starvation, this situation is per se capable of inducing GS in macrophages, CAFs, adipocytes and T cells. In these cells GS is known to modify significantly their metabolic and functional phenotype in a protumoral direction due to the acquired glutamine-synthesizing property (Figure 2). In this crosstalk, GS versus GLS expression in cancer cells could play a crucial role.

Glutamine catabolism is among the targets for development of anticancer drugs. However, this strategy alone has shown limited efficacy. Evidence on the crosstalk between cancer and stromal cells highlights a totally new scenario in which cancer cell metabolism influences stromal cells metabolism and function, to the point that immunometabolic targets are emerging for drug discovery. A combination therapy targeting glutamine metabolism in both cancer and stromal cells holds a promising future to achieve a valuable approach for therapeutic success. For its strong significance with respect to the stromal cell phenotype, especially its immune component, glutamine synthesis represents a key metabolic step since (i) inhibition of GS activity has been shown to induce a strong metabolic and functional reprogramming to be exploited against cancer and metastasis; (ii) the ability to synthesize GS by stromal cells is easily manipulable by cancer cells in virtue of their glutamine dependency due to lack of GS. A reduced glutamine synthesizing capacity in cancer cells might be one of the elements driving GS expression in the cells of the TME, which favors immunosuppression and supplies glutamine. This indicates that the significance of GS expression within tumors is cell specific and the comprehension of its functional role needs to take into account the different and conflicting expression levels of GS within the different malignant and non-malignant cellular players in the tumor. Additional comprehensive investigations on other key metabolic targets and on how they shape stromal cell function are strongly awaited.

## Figures and Tables

**Table 1:** The complexity of glutamine synthetase (GS) expression in cancer cells.

Tumor Type	GS Expression	Phenotype	References
Breast (luminal) Breast Breast (basal)	High	Low aggressiveness and therapy-resistance	[71]
	High	(HER2^+^, ER^+^) High aggressiveness	[79,80]
	Low	High aggressiveness and therapy-resistance	[71]
Liver	High	(MET-induced) Glutaminase inhibition-resistance	[81]
	High	Sorafenib sensitivity	[69]
	Low	Sorafenib resistance	[69]
	Low	(MYC-induced) Glutaminase inhibition-sensitivity	[81]
Glioblastoma multiforme	High	High aggressiveness, Glutaminase inhibition-resistance	[67]
Non-Small Cell	High	(MYC-induced) Glutaminase inhibition-resistance	[81]
Lung Carcinoma	Low	Glutaminase inhibition-sensitivity	[75]
Ovary	High	(CD90^+^ cancer stem-like cells) High tumorigenicity	[83]
	High	Low invasiveness	[73]
	Low	High invasiveness	[73]
Melanoma	High	Cytotoxic T lymphocyte killing sensitivity	[72]
	Low	Cytotoxic T lymphocyte killing resistance	[72]

ER: estrogen receptor; HER2: human epidermal growth factor receptor 2; CD90: cluster of differentiation 90.
